# Fabrication Process and Particle Dispersion Characteristics of W–PETG-Based 3D-Printed Composites for Medical Radiation Shielding

**DOI:** 10.3390/polym18020268

**Published:** 2026-01-19

**Authors:** Seon-Chil Kim

**Affiliations:** Department of Biomedical Engineering, School of Medicine, Keimyung University, 1095 Dalgubeol-daero, Daegu 42601, Republic of Korea; chil@kmu.ac.kr; Tel.: +82-10-4803-7773

**Keywords:** radiation shielding, 3D printing, W–PETG composite, syringe shield, particle dispersion

## Abstract

In this study, a W–polyethylene terephthalate glycol (PETG)-based 3D-printed composite was designed for medical radiation shielding, and syringe shielding components were fabricated to evaluate shielding performance and particle dispersion characteristics. Up to 70 wt% of tungsten powder was incorporated into the PETG polymer matrix to produce W–PETG filaments suitable for 3D printing. Using the fused deposition modeling (FDM) method, a 3.0 mm-thick radiation shielding cover for a 10 mL syringe was fabricated. Radiation shielding performance was assessed using a ^99m^Tc (200 µCi) source at distances of 30, 50, and 100 cm. While a conventional 1.0 mm Pb shield exhibited shielding efficiencies of 92.24%, 94.26%, and 95.13% at each distance, the 3.0 mm W–PETG shield demonstrated efficiencies of 70.67%, 75.64%, and 77.57%, respectively. Higher temperatures improved shielding efficiency by approximately 5.48 percentage points. When processed above 160 °C, tungsten particle clustering decreased and a more uniform dispersion was achieved, enhancing shielding performance. The interrelationship among filament fabrication parameters, particle dispersion behavior, and shielding performance of W–PETG composites was quantitatively demonstrated. The lightweight, geometric design flexibility, and compatibility with 3D-printing processes of W–PETG composites suggest strong potential as alternative materials for custom medical radiation shielding devices.

## 1. Introduction

The expansion of radiation-based diagnostic and therapeutic technologies in the medical field has driven the development of compact and mobile radiological diagnostic equipment [1]. Simultaneously, medical institutions maintain strict control over radiation-generating areas designated as controlled protection zones, and a range of shielding technologies and devices have been developed and implemented. Currently, most radiation-shielding materials used in medical settings remain primarily lead-based and are implemented in multiple forms, including protective garments, barriers, mobile partitions, and specialized components within medical devices [2]. Although lead provides robust shielding performance and favorable processability, increasing concerns regarding environmental toxicity, excessive weight, limitations in thickness control, limited durability, and industrial hygiene issues during manufacturing, use, and disposal have intensified the demand for environmentally sustainable metallic composite materials capable of replacing lead [3].

High-atomic-number metals such as tungsten (W) have been actively investigated as alternative radiation-shielding materials [4]. For metal-based shields, manufacturability is as important as the shielding performance, particularly for medical components that require lightweight designs based on precise weight control and fabrication into complex geometries compatible with device structures [5]. In addition, reliable shielding performance depends on appropriate particle-packing density, effective control of pinholes, and minimization of internal voids during fabrication [6]. Therefore, radiation-shielding components require optimal shielding efficiency as well as advanced manufacturing processes capable of reproducibly achieving the desired geometry and performance.

Conventional methods for manufacturing radiation shields typically involve the fabrication of films, sheets, or membranes by mixing polymers with metal particles to improve the flexibility of metallic materials and enhance user convenience [7]. However, strength reduction and increased weight associated with higher metal-particle loading remain major challenges. These strength-to-weight trade-offs are closely related to particle density, particle-size distribution, polymer–metal interfacial bonding, and cross-linking characteristics of the composite materials [8]. Because the shielding performance depends fundamentally on the atomic number, density, and arrangement of the shielding material [9], performance improvements within a given category of materials require advanced process technologies such as particle size reduction (nano- or micro-scale powderization), composite-structure design, surface modification of powder particles, and layered-architecture engineering [10]. To achieve targeted shapes and dimensions, polymer–metal powder composites are typically processed employing extrusion molding, calendering, or die-molding techniques [11,12].

However, although these techniques facilitate mass production of uniform geometries, they are inefficient for fabricating small quantities of customized components. Clinical applications increasingly demand compact and irregularly shaped radiation-shielding components, including specialized internal parts of medical devices and tools designed to selectively shield specific anatomical regions during diagnostic or interventional procedures [13]. In particular, gamma-ray inspection devices utilizing radioactive isotopes often require localized shielding in diverse geometries depending on the intended use and structural configuration of the equipment; syringe-shield components represent a widely used example [14]. These components are subject to significant geometric constraints and diverse user-specific requirements, rendering conventional mold-based or casting processes inadequate for efficient supply.

Given these limitations, this study proposes a manufacturing process that enables customized design and fabrication of medical radiation-shielding components via 3D printing (additive manufacturing) technology. Although 3D printing is already used extensively for the fabrication of medical devices and implants owing to its suitability for small-batch, multivariant manufacturing [15], its application to radiation-shielding structures based on metal–polymer composite filaments remains in the early stages of development. The key for 3D-printed radiation shields involves achieving reproducible shielding performance, which depends on the particle dispersion characteristics, specifically the degree of uniform distribution of shielding metal particles within the composite matrix [16]. Although finer metal particles offer improved dispersibility and therefore potential enhancement of shielding performance, they simultaneously introduce issues such as increased viscosity and reduced extrusion stability, which complicate the injection process control and hinder the reproducibility of mechanical strength [17]. Therefore, filament fabrication for 3D printing requires optimized process parameters, including polymer material selection, metal loading fraction, and quantitatively controlled mixing and extrusion conditions, to achieve adequate shielding performance and manufacturability [18].

In this study, polyethylene terephthalate glycol (PETG) was selected as the polymer matrix, and tungsten (W) powder was incorporated to produce W–PETG composite filaments for 3D printing applications [19]. Using these filaments, syringe-shielding components designed for protection during radioactive isotope-based vascular injection procedures were fabricated via 3D printing, and their shielding performance was evaluated in comparison with that of conventional lead-based shielding devices. Notably, syringe shields, which are widely used in medical institutions, are manufactured primarily via lead-based casting processes; therefore, they represent a promising application area for replacement with 3D printing owing to their geometric complexity and functional requirements.

Beyond the fabrication of prototype components, this study also investigates the correlation between manufacturing-process parameters for W–PETG composite filaments and particle-dispersion characteristics.

Based on these findings, this study introduces a processing strategy for radiation-shielding composite materials that enable 3D printing by combining environmentally friendly metallic shielding materials with a polymer matrix. The results provide foundational data for the development of lightweight, geometrically versatile, and high-performance shielding components applicable across a broad range of industries requiring radiation protection.

Radiation-shielding materials play a critical role across multiple fields, including the medical sector, aerospace, nuclear engineering, and industrial radiography [20]. Research on the fabrication process and particle-dispersion characteristics of W–PETG-based 3D-printable composites, which are capable of overcoming the limitations of lead-based shields while enabling complex geometries and customized designs, offers an essential foundation for advancing next-generation medical radiation-shielding devices.

## 2. Methods and Materials

The attenuation of X-rays and γ-rays transmitted through a shielding medium depends directly on the material’s linear attenuation coefficient (μ; mass attenuation coefficient) and the thickness (χ) of the shielding layer [21]. Therefore, the transmitted photon intensity I relative to the incident photon intensity ${Ι}_{0}$ is expressed in Equation (1) [22]. The linear attenuation coefficient can be described using Equation (2), and the mass attenuation coefficient ($\mu_{m}$) can be expressed as Equation (3), where $\omega_{i}$ is the density of the material and ρ represents the elemental density [23]. Here, $\omega_{i}$ and ${\left(\frac{\mu }{\rho }\right)}_{i}$ denote the mass fraction and the mass attenuation coefficient of the *i*-th constituent element, respectively.

(1) $$I=I_{0}exp(-\mu x)$$

(2) $$\mu ({cm}^{-1})=\frac{1}{t}ln(\frac{I}{I_{0}})$$

(3) $$\mu_{m}=\sum_{i}\omega_{i}({\frac{\mu }{\rho })}_{i}$$

The relationship between the linear attenuation coefficient (μ) and the mass attenuation coefficient ($\mu_{m}$) is expressed by Equation (4) [24]. The total mass attenuation coefficient of an elemental mixture in a composite material (μ/ρ) can be described by Equation (5) [25].

(4) $$\mu =\mu_{m}\times \rho$$

(5) $$\mu /\rho =\sum_{i}\omega_{i}{\left(\frac{\mu }{\rho }\right)}_{i}$$

Therefore, when incident radiation passes through a shielding material, interactions with the internal atoms reduce the initial photon intensity. Maximizing this attenuation requires selecting materials with high atomic numbers or increasing both the thickness and density of the shielding structure during fabrication [26].

In this study, tungsten was selected as the metallic shielding material. Tungsten, with an atomic number of 74 and a density of 19.3 g/cm^3^, exhibits excellent gamma-ray absorption characteristics among high-density metals [27]. For gamma rays in the energy range of several hundred keV or higher, tungsten effectively attenuates the energy via photoelectric absorption and Compton scattering. Additionally, its high melting point (3422 °C) and outstanding mechanical stability make it suitable as a filler material for composite shielding structures [28]. The polymer matrix selected in this study was polyethylene terephthalate glycol (PETG), a modified form of PET wherein glycol improves thermal resistance (approximately 80 °C) and processability. PETG provides high flexibility and is suitable for mixing with metal powders, thereby enabling high-density micrometal particle loading [29]. Its low hygroscopicity stabilizes the final fabricated components. In addition, the tensile strength of PETG-based 3D-printed specimens has been reported to be in the range of 46–50 MPa [30]. However, polymer aging induced by radiation absorption may lead to surface hardening; nevertheless, such effects require a prolonged period before they can significantly influence the shielding performance.

Initially, to increase the tungsten content in the base W–PETG composite, PETG and tungsten were premixed in powder form and processed into pellets. The fabricated pellets were extruded using a screw extruder to produce filaments with a diameter of 1.75 ± 0.03 mm. As the tungsten content increased, the extrusion flow in the screw extruder decreased, resulting in reduced filament processability. Therefore, 70 wt% was identified as the optimal mixing ratio; a higher tungsten content caused clogging at the 0.4 mm nozzle inlet. Therefore, the tungsten content was limited to 70 wt% to ensure mechanical stability and strength after extrusion.

The extrusion temperature of the filament fabrication extruder (Ningbo, KED 2680, China, 2018) was set in the range of 180–210 °C, and the extrusion speed was between 25 and 50 rpm. Although increasing the tungsten content improves shielding performance, extrusion stability deteriorates due to increased melt viscosity and particle agglomeration, which can induce stagnation or clogging at the nozzle entrance, thereby requiring a reduction in extrusion speed [31]. The decrease in extrusion speed is associated with the relationship between the nozzle inlet diameter and effective size of the agglomerated tungsten particles. In the present study, when a screw diameter of 25D and a nozzle diameter of 0.4 mm were employed, the maximum stable screw rotation speed was maintained at 50 rpm at a tungsten content of 70 wt%. 

**Figure 1:**
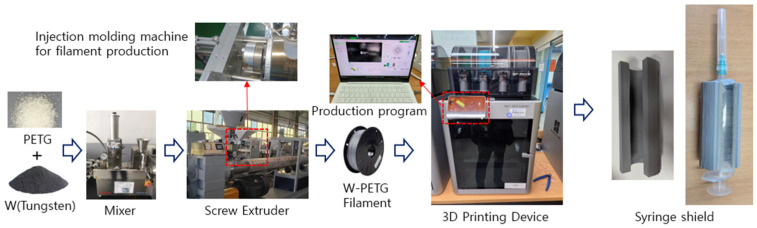
3D printing syringe shield manufacturing process.

3D printing was performed using a Bambu Lab X1 Carbon printer (PF030-D, Shenzhen Bambu Lab Technology Co., Ltd., Shenzhen, China, 2023). The exposure temperature was set between 120 and 180 °C to secure mechanical strength with the fused deposition modeling (FDM) process, and the printing was performed with an increased thickness of 0.2 mm, following the procedure shown in Figure 1. The final printed syringe shield had a thickness of 3.0 mm, and its design geometry replicated that of a conventional lead-based syringe shield used in clinical practice [32].

**Figure 2:**
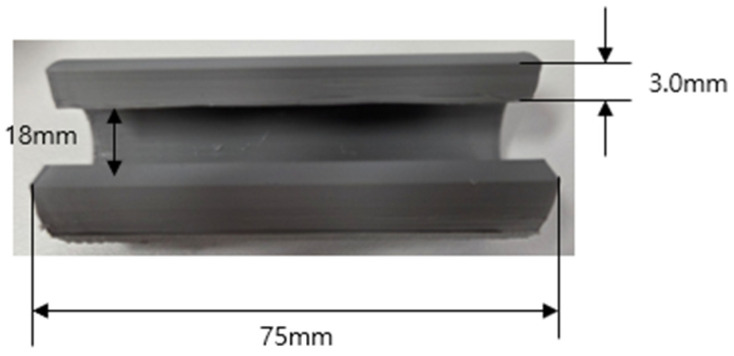
Fabricated syringe shield.

In this experiment, a syringe shield was fabricated using 3D printing, as shown in Figure 2. The final product was manufactured to accommodate a 10 cc syringe, with an inner diameter of 18 mm, a length of 75 mm, and a thickness of 3.0 mm. Although the conventional lead-based syringe shield weighs 450 ± 1.25 g, the 3D-printed shield produced in this study weighs 280 ± 1.2 g. In addition, the dispersion state of the tungsten particles in the experimental specimens was visually examined using a field-emission scanning electron microscope (FESEM; Hitachi S-4800, Hitachi High-Tech, Tokyo, Japan) [33].

The radiation source used in this experiment was ^99m^T_C_, and its characteristics are summarized in Table 1 [34]. The radioisotope ^99m^Tc (200 μCi) emits gamma rays with a photon energy of 140.5 keV and is among the most widely used radionuclides in nuclear medicine imaging, including bone scintigraphy and organ function evaluation [35]. Therefore, it was selected for the shielding performance comparison experiments with lead. The activity of 200 μCi corresponds to a relatively low dose, which is typically administered for pediatric bone scanning [36]. ^99m^Tc has a half-life of 6.02 h and exhibits rapid radioactive decay, making it suitable for radiation-shielding research and medical diagnostic applications [37].

**Figure 3:**
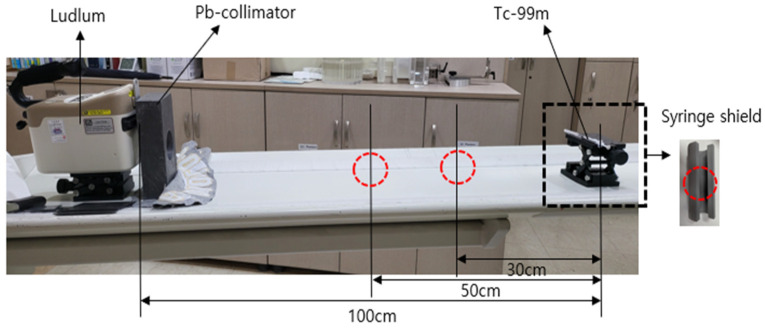
Geometric configuration for gamma-ray shielding evaluation.

The experimental setup used to evaluate the gamma-ray shielding performance of the fabricated material is shown in Figure 3. The radiation dose was measured using an isotope identifier with an internal detector (Ludlum Model 702i, Ludlum Measurements, Inc., Sweetwater, TX, USA), which recorded the transmitted gamma ray intensity after passing through a sample-shielding structure [38]. The geometric configuration for the radiation measurement was established by setting the distance between the syringe shield containing the radiation source and the detector to 100 cm, as illustrated in Figure 3. The detector was equipped with an internal NaI detector (5.1 cm × 3.8 cm), and measurements were conducted for approximately 60 s. For comparison, the shielding performance of a conventional lead syringe shield fabricated under the same measurement conditions was also evaluated [39].

The shielding performance of the syringe shield was evaluated by comparing the gamma ray dose before and after the transmission of ^99m^Tc, the radioactive isotope. The transmitted dose (D_lu_) was measured with and without the fabricated shield to determine the change in transmitted radiation. The shielding rate was calculated using Equation (6). The shielding performance was measured 10 times at varying distances, and the average values were reported [40].

(6) $$Radiation\;shielding=1-(\frac{D_{lu}\mathrm{with}\;\mathrm{shielding}}{D_{lu}\;\mathrm{without}\;\mathrm{shielding}})\times 100(\%)$$

## 3. Results

**Figure 4:**
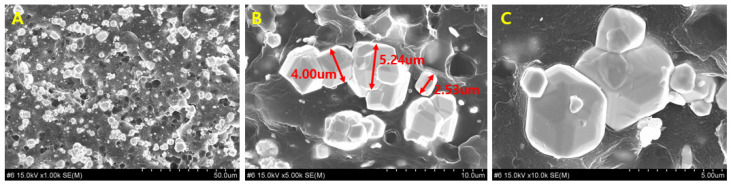
Cross-sectional particle analysis of the molded shielding sample: (**A**) Dispersion state of tungsten particles within the overall PETG polymer matrix; (**B**) formation of tungsten particle clusters within the PETG region; (**C**) smaller tungsten particles attached around larger tungsten particles.

The dispersion of tungsten particles within the syringe shield fabricated employing the W–PETG composite was observed under high magnification, as shown in Figure 4. In Figure 4A, tungsten particles are clearly visible within the 3.0 mm-thick shield and display uniform distribution within the PETG polymer matrix without prominent particle agglomeration, indicating the presence of individually dispersed particles.

Uniform particle distribution in the 3D-printed shielding structure is highly dependent on the filament fabrication process. In Figure 4A, the metal particles appear evenly spaced within the matrix. However, Figure 4B, a magnified image of Figure 4A, shows localized particle clustering within the PETG matrix, and the further magnified image in Figure 4C shows smaller particles located around larger tungsten particles.

This phenomenon represents a critical challenge in the FDM process used for 3D printing because particle agglomeration can influence the shielding performance owing to entrapped air layers or cooling-related irregularities. Therefore, in this study, the 3D printing process was performed at temperatures exceeding 160 °C, and Figure 4 confirms the absence of air-layer intrusion under these conditions.

The shielding performance of the W–PETG composite fabricated by 3D printing was evaluated. The gamma-ray shielding performance of the W–PETG syringe shield was compared with that of a conventional lead syringe shield, as summarized in Table 2. At a distance of 100 cm, the shielding performance of the fabricated W–PETG shield was 17.56% lower than that of the 1.0 mm lead syringe shield. At a distance of 30 cm from the ^99m^Tc source, the W–PETG syringe shield provided a shielding efficiency of approximately 70%. A higher tungsten content in the composite is required to further reduce the performance gap relative to that of lead.

**Figure 5:**
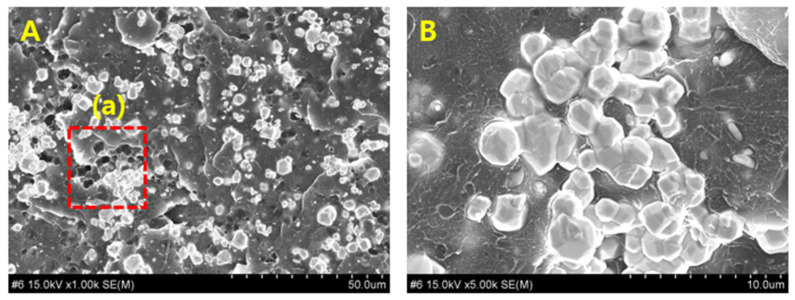
Particle clustering behavior within the shielding composite: (**A**) Tungsten particle distribution within the overall PETG polymer matrix, and (**B**) magnified view of region (a), showing clustered tungsten particles.

**Figure 6:**
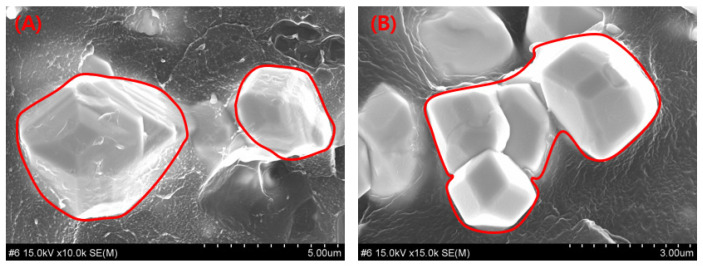
Enlarged cross-sectional images of the syringe shield fabricated utilizing the W–PETG composite: (**A**) Magnified view showing uniform dispersion of tungsten particles, and (**B**) magnified view showing cluster formation resulting from poor dispersion and agglomeration of tungsten particles.

During melt injection and extrusion in confined spaces, metallic particles agglomerate within the filament material, as shown in Figure 5A. As illustrated in the magnified image in Figure 5B, such particle clustering can induce a streaming effect wherein radiation travels straight through the polymer regions devoid of shielding particles, thereby reducing the overall shielding performance [41]. The analysis of the tungsten particle clustering behavior observed in Figure 5 indicates that its origin can be attributed to thermal control issues during the extrusion and 3D printing processes [42]. Therefore, experiments were conducted by controlling the extrusion/printing temperature, and the results are shown in Figure 6. The samples in Figure 6A,B were printed at 160 ± 10 °C and 120 ± 10 °C, respectively. More pronounced tungsten particle clustering and formation of tungsten agglomerates are observed in Figure 6B than in Figure 6A. These results indicate the potential of controlling metal particle clustering by adjusting the thermal conditions of the W–PETG filament during 3D printing.

The influence of extrusion temperature control during the 3D printing process on the shielding performance of the syringe shields was evaluated, as summarized in Table 3. A comparison of shielding efficiency between low- and high-temperature 3D-printed specimens showed prominent differences. At a distance of 100 cm, the shielding-performance difference reached 5.48%, with higher printing temperatures yielding superior shielding effectiveness. This improvement can be attributed to a reduction in particle clustering.

## 4. Discussion

Radiation-shielding structures can generally be classified into flexible sheet-type and rigid shields, which form barrier walls based on their structural strength. The flexible sheet-type shields are typically fabricated by mixing polymer materials with shielding fillers and processing the composites via calendering to impart flexibility. By contrast, the rigid shields are primarily produced from metallic materials designed to maintain a certain thickness and mechanical strength [43,44]. In composite systems, the shielding performance directly depends on the uniformity of dispersion of the shielding metal particles within the polymer matrix [45].

In contrast to conventional fabrication approaches such as calendering, molding, and extrusion, the present study proposes a new manufacturing process that employs 3D printing to directly design and fabricate compact shielding tools used in medical institutions. The key requirement for 3D-printing-based shielding components is the reproducibility of their shielding performance, which is governed by the uniform dispersion of tungsten particles in the shielding composite [46]. Uniform dispersion in a polymer matrix is highly sensitive to mean particle size and particle size distribution [47]. In this study, microsized tungsten particles of different sizes were employed to achieve a dense packing effect, whereby smaller particles filled the voids between larger particles.

However, during the actual fabrication of filaments for 3D printing, cluster formation owing to particle agglomeration was observed, resulting in local regions with effectively larger particle sizes. This phenomenon can be attributed to insufficient thermal control of the polymer matrix during extrusion. Ideally, the polymer matrix must remain in a molten state within an appropriate viscosity range to ensure adequate particle mobility and allow rearrangement and redispersion within the matrix. Concurrently, because the material must be supplied in filament form, identifying an appropriate viscosity–temperature window that ensures adequate particle mobility and dimensional stability of the filament is critical.

For W–PETG, sufficient flowability to promote tungsten particle dispersion is achieved at temperatures exceeding approximately 160 °C. This indicates that appropriate thermal control during filament fabrication can facilitate the rearrangement and redispersion of microscale metal particles, thereby substantially mitigating particle agglomeration and cluster formation. Therefore, this study demonstrates that thermal and rheological control during the filament extrusion stage is a critical processing parameter that governs the internal microstructure and shielding performance of W–PETG-based shielding components. The experimental results further indicate that the shielding performance of 3D-printed components fabricated from W–PETG composites can be improved by suppressing tungsten particle clustering and achieving a more uniform dispersion within the matrix. Accordingly, from a process engineering perspective, sufficient dry mixing of tungsten powder with polymer pellets prior to filament extrusion, followed by a thermally controlled extrusion process, is recommended. Such process optimization is expected to promote uniform particle dispersion and is crucial in ensuring both the shielding performance and reproducibility of the 3D-printed shielding components.

Conventional manufacturing processes for shielding structures have predominantly evolved to support mass production and repetitive fabrication of uniform geometries [48]. However, in clinical practice, the demand for custom-shaped shielding tools has increased owing to variations in patient conditions, examination or procedure sites, and radiation access patterns of medical personnel [49]. The production of such specialized shielding tools using traditional manufacturing methods requires complex mold designs, long lead times, and substantial costs. Although castable and machinable lead has been considered as an alternative in the past, its use and disposal pose significant environmental, occupational, and human health hazards owing to heavy metal toxicity [50]. Consequently, the demand has grown for alternative materials capable of replacing lead and manufacturing technologies capable of flexible shaping and fabrication.

In this context, the proposed W–PETG-based 3D-printing method provides a practical alternative technology that enables medical institutions to fabricate small application-specific shielding tools rapidly and with relative simplicity. Furthermore, by analyzing the dispersion characteristics of structural shielding fillers within the filaments and discussing the process conditions that influence these characteristics, this study offers valuable foundational insights for achieving reproducible shielding performance in the final 3D-printed products.

To appropriately control particle dispersion within 3D-printed composites, it is crucial to ensure sufficient mobility of metal particles during the melt injection and extrusion stages. In the subsequent printing and layer stacking stages, particle rearrangement driven by mechanical shear and thermally induced redispersion mechanisms within the polymer matrix must be incorporated. Therefore, securing optimal mixing temperatures, residence times, and extrusion conditions tailored to the composition of the shielding material is a key technological requirement for fabricating high-performance 3D-printed shielding components.

This study introduces a practical and concrete method for fabricating the radiation-shielding components required in medical institutions employing relatively simple equipment and processing steps, thereby substantially improving the accessibility of shielding manufacturing technologies. One limitation of this study is that it focused on blending a single metallic filler, tungsten, with a polymer matrix; therefore, the interactions and particle property variations inherent to multicomponent metal composites were not investigated. In addition, the evaluation of shielding performance was based solely on a single-energy gamma-ray source (^99m^Tc, 140.5 keV), and thus a systematic analysis across a broader energy spectrum of other radionuclides—such as ^131^I, which emits gamma rays at approximately 364 keV, and ^68^Ga, which emits gamma rays at approximately 184 keV—has not been conducted. Nevertheless, by presenting the shielding performance of standard lead as a reference, the results of this study can be expressed in terms of lead equivalence, thereby providing a meaningful basis for comparison with conventional shielding materials. This study experimentally demonstrated that applying 3D-printing technology to the fabrication of radiation-shielding components is a realistic approach and represents a promising strategy for replacing or complementing conventional lead-based shielding.

In future research, multimaterial composite filaments incorporating various metal powders will be developed, and their energy-dependent shielding characteristics and long-term durability and clinical applicability will be investigated [51]. Based on such follow-up studies, 3D-printing-based radiation shielding technology can establish itself as a customized, environmentally friendly, and high-performance shielding solution across the medical and industrial fields, and it can be regarded as an area that warrants sustained research and development.

## 5. Conclusions

Using W–PETG-based 3D-printed composites, a syringe-type radiation shielding component was fabricated. The 3.0 mm-thick W–PETG shield was approximately 38% lighter than the conventional 1.0 mm lead shield, while providing a maximum shielding efficiency of approximately 79% against ^99m^Tc (140.5 keV) gamma rays. When the extrusion and printing temperatures were controlled above 160 °C, tungsten particle clustering was reduced and particle dispersion was improved, enhancing shielding performance by more than 5 percentage points. These findings indicate that, although W–PETG exhibits somewhat lower shielding efficiency than lead, it may serve as a promising alternative technology for customized medical radiation shielding components when considering its advantages in weight reduction, geometric freedom, and process flexibility.

## Figures and Tables

**Table 1:** Characteristics of the nuclear medicine radiation source.

Characteristic		Radionuclide (Unit: keV)
		^99m^Tc
Half-life		6.02 h
Emitting Energy	γ emission	140.5 (0.89)
		18.4 (0.04)
		18.3 (0.02)

**Table 2:** Gamma-ray shielding effectiveness using ^99m^Tc: Comparison between Pb and W–PETG.

Shielding Material	Dose Rate (mR/h) *			Shielding Rate (%)		
	30 cm	50 cm	100 cm	30 cm	50 cm	100 cm
-	19.859	8.723	3.121	0	0	0
Pb (1.0 mm)	1.542 ± 0.002	0.501 ± 0.002	1.152 ± 0.001	92.24	94.26	95.13
W-PEGT (3.0 mm)	5.824 ± 0.004	2.125 ± 0.001	0.700 ± 0.002	70.67	75.64	77.57

* Dose rates are presented as mean ± standard deviation.

**Table 3:** Comparison of gamma-ray shielding performance according to 3D printing extrusion temperature using ^99m^Tc.

W-PEGT 3.0 mm	Dose Rate (mR/h) *			Shielding Rate (%)		
	30 cm	50 cm	100 cm	30 cm	50 cm	100 cm
-	19.859	8.723	3.121	0	0	0
120–160 °C	6.120 ± 0.001	2.451 ± 0.002	0.825 ± 0.010	69.18	71.90	73.57
160–180 °C	5.874 ± 0.001	2.145 ± 0.002	0.654 ± 0.003	70.42	75.41	79.05

* Dose rates are presented as mean ± standard deviation.

## Data Availability

All data generated during this study are included in this manuscript.
